# Editorial: Functional approach to neurosurgery: current research and future perspectives

**DOI:** 10.3389/fsurg.2024.1533526

**Published:** 2024-12-18

**Authors:** Nicola Montano, Renata Martinelli, Quintino Giorgio D’Alessandris, Alessandro Izzo, Manuela D’Ercole

**Affiliations:** ^1^ Department of Neuroscience, Neurosurgery Section, Università Cattolica del Sacro Cuore, Rome, Italy; ^2^ Department of Neurosurgery, Fondazione Policlinico Universitario Agostino Gemelli IRCCS, Rome, Italy

**Keywords:** functional neurosurgery, functional neuro-oncology, vascular surgery, spine surgery, artificial intelligence, intraoperative neuromonitoring

**Editorial on the Research Topic**
Functional approach to neurosurgery: current research and future perspectives

In recent years, neurosurgery has shifted toward a “functional approach,” emphasizing the preservation of neurological function in every procedure. Traditionally, functional neurosurgery involved modulating the nervous system to manage conditions such as movement disorders, chronic pain, or epilepsy. However, this approach has expanded into other domains: in neuro-oncology, preserving postoperative function is associated with improved survival rates in patients with both lower grade and malignant gliomas, and the broad use of intraoperative neuro-monitoring (IONM) and brain mapping techniques enables safer tumor resections. Routine use of IONM in vascular and spine surgery has also improved functional outcomes. Advances in artificial intelligence, machine learning, and connectomics further enhance the potential for understanding neural networks and improving neurosurgical outcomes.

This special issue highlights the importance of innovations stemming from the functional approach, showcasing its applicability across various fields. Pérez-Cruz et al. emphasize the significance of enhancing preoperative training for the next generation of neurosurgeons. They propose a new technique involving latex injection of the vascular system in brain and head-neck segments in cadaveric specimens, replacing resins and silicone for microvascular study and training (1, 2). This approach allows for greater realism in vascular perfusion, enabling improved visualization of small vessels, and offers potential benefits in areas like microvascular decompression training, skull base surgery simulation, and endoscopic skills development.

In the quest to optimize the quality and utilization of IONM—now essential for many neurosurgical procedures—Yang et al. evaluated the effect of dexmedetomidine, often used as an adjunct to total intravenous anesthesia (TIVA), on the risk of masking neurological deficit warnings in intraoperative monitoring (3, 4). They particularly assessed its effects on transcranial motor-evoked potentials (TcMEPs) and somatosensory-evoked potentials (SSEPs). The authors demonstrated that dexmedetomidine does not significantly impact motor and sensory outcomes in patients undergoing craniotomy, as there is no statistical association between its use in patients undergoing IONM and changes in SEPs and MEPs. However, an association was found with a decrease in TcMEPs, although a similar decrease was observed in the contralateral non-operated side. This finding does not affect surgical outcomes but aids in interpreting potential bilateral intraoperative decreases in TcMEPs.

Two articles focused on investigating the benefits of utilizing innovative tools and surgical techniques in non-conventional yet functionally effective ways. Battistelli et al. described two cases in which Wiltse approach, a traditional anatomical approach to the far lateral spinal compartment conducted via dissection of the medial multifidus and lateral longissimus muscles (5, 6), was performed with O-arm CT neuronavigation and IONM. A left lumbar intra-extraforaminal schwannoma and an extraforaminal lumbar disc herniation at the adjacent level of previous lumbar instrumentation were operated in this way. The authors noted several advantages, including minimal intraoperative bleeding, shorter hospital stays, and low infection rates compared with a standard midline approach. As with any CT-neuronavigation procedure, patient reference placement is a critical error source: it should be positioned on the nearest spinous process relative to the surgical level to enhance navigation accuracy, reinforcing its significant utility in spinal pathology. Lu et al. proposed the use of endoscopy in the surgical management of intracerebral hematomas, introducing a new minimally invasive endoscopic removal technique for intracerebral hemorrhage. This technique employs a novel contact endoscope to assist surgeons in locating the hematoma under direct vision through a portable and contact neuro-endoscope (7). The direct visualization allows for minor adjustments to the puncture direction during the operation, ensuring precise positioning and reducing the risk of blood vessel puncture and deviation in the puncture site.

In some cases, a functional approach in neurosurgery can improve the prediction of the disease outcomes. Sun et al. demonstrated this fact by examining brain volume with 3D-Slicer software at two time points post-hemorrhage (within the first 24 h and between 24 and 48 h after aneurysmal subarachnoid hemorrhage, aSAH). They found that elevated brain swelling was more strongly associated with delayed cerebral ischemia (DCI) in the group experiencing swelling than in the non-swelling group (8). Additionally, the risk association was directly proportional. This study suggests that the swelling rate of brain volume (SRBV) could serve as a surrogate numerical indicator of early brain injury (EBI) in the initial phase, potentially predicting DCI occurrence. Higher SRBV in the early stages of aSAH was linked to increased DCI incidence compared with lower SRBV.

Finally, Battistelli et al. conducted a review demonstrating that artificial intelligence (AI) is emerging as an increasingly widespread tool in medicine for enhancing diagnostic accuracy, treatment selection, and drug development (9, 10). Its application to pathologies with poorly understood etiologies, such as trigeminal neuralgia, could be pivotal in creating algorithms that guide surgical indications in a personalized manner. As the authors highlighted, the development of software dedicated to trigeminal neuralgia is already enabling us to obtain critical preoperative data on the role of neurovascular conflict, and future research appears promising in defining its role in prognosis estimation and recurrence predictability by combining radiomics data with clinical findings and data collected intraoperatively through intraoperative monitoring.
